# Machine Learning-Based Identification of Potentially Novel Non-Alcoholic Fatty Liver Disease Biomarkers

**DOI:** 10.3390/biomedicines9111636

**Published:** 2021-11-07

**Authors:** Roshan Shafiha, Basak Bahcivanci, Georgios V. Gkoutos, Animesh Acharjee

**Affiliations:** 1 Centre for Computational Biology, Institute of Cancer and Genomic Sciences, University of Birmingham, Birmingham B15 2TT, UK; rxx063@student.bham.ac.uk (R.S.); basakbahcivanci@gmail.com (B.B.); g.gkoutos@bham.ac.uk (G.V.G.); 2 Institute of Translational Medicine, University of Birmingham, Birmingham B15 2TT, UK; 3 NIHR Surgical Reconstruction and Microbiology Research Centre, University Hospital Birmingham, Birmingham B15 2WB, UK; 4 MRC Health Data Research UK (HDR UK), Midlands Site, Birmingham B15 2TT, UK; 5 NIHR Experimental Cancer Medicine Centre, Birmingham B15 2TT, UK; 6 NIHR Biomedical Research Centre, University Hospital Birmingham, Birmingham B15 2TT, UK

**Keywords:** NAFLD, biomarker, machine learning, transcriptomics, lipidomics

## Abstract

Non-alcoholic fatty liver disease (NAFLD) is a chronic liver disease that presents a great challenge for treatment and prevention.. This study aims to implement a machine learning approach that employs such datasets to identify potential biomarker targets. We developed a pipeline to identify potential biomarkers for NAFLD that includes five major processes, namely, a pre-processing step, a feature selection and a generation of a random forest model and, finally, a downstream feature analysis and a provision of a potential biological interpretation. The pre-processing step includes data normalising and variable extraction accompanied by appropriate annotations. A feature selection based on a differential gene expression analysis is then conducted to identify significant features and then employ them to generate a random forest model whose performance is assessed based on a receiver operating characteristic curve. Next, the features are subjected to a downstream analysis, such as univariate analysis, a pathway enrichment analysis, a network analysis and a generation of correlation plots, boxplots and heatmaps. Once the results are obtained, the biological interpretation and the literature validation is conducted over the identified features and results. We applied this pipeline to transcriptomics and lipidomic datasets and concluded that the C4BPA gene could play a role in the development of NAFLD. The activation of the complement pathway, due to the downregulation of the C4BPA gene, leads to an increase in triglyceride content, which might further render the lipid metabolism. This approach identified the C4BPA gene, an inhibitor of the complement pathway, as a potential biomarker for the development of NAFLD.

## 1. Introduction

Non-alcoholic fatty liver disease (NAFLD) is a form of chronic liver disease that affects 20–30% of the western population and approximately 25% of the global population [1,2,3] NAFLD is associated with a wide range of diseases, including increased visceral obesity and metabolomic abnormalities, such as insulin resistance, diabetes, hypertension, dyslipidemia, atherosclerosis and systemic micro-inflammation [4,5,6,7,8,9]. Currently, enhanced by an inactive lifestyle and unhealthy food culture, the spread of NAFLD has increased across countries among different age groups [4,10]. The disease has increased from 15% in 2005 to 25% in 2010 with a subsequent increase in the number of obesity cases [11]. It is also anticipated that there will be an increase in the number of NAFLD cases from 83.1 million (2015) to 100.9 million (2030) [12].

NAFLD consists of a spectrum of hepatic abnormalities ranging from steatosis or non-alcoholic fatty liver to the various levels of necrotic inflammation leading to non-alcoholic steatohepatitis (NASH) [13]. The minority of NAFLD cases progresses to liver disease complications resulting to 4–8% deaths from cirrhosis complications and 1–5% deaths from hepatocellular carcinoma [11]. The initial stages of NAFLD, characterised by complex pathogenesis, include the accumulation of triglycerides in hepatocytes, which might further develop to conditions such as inflammation, fibrosis and cellular death, which are characteristics of NASH [13]. It is considered that there are multiple factors which might lead to NAFLD [13,14]. NAFLD risk factors include an unhealthy diet and an inactive lifestyle and it is expected that the interaction between the genetic characteristics, diet and gut microbiota of an individual play a pivotal role in our understanding of the development and progression of the disease.

Understanding the pathogenesis of NAFLD will cater a better understanding of the pathophysiological processes underlying the disease and will likely highlight potential therapeutic interventions [4]. While the molecular mechanism, involved in the addition of fats in the liver, is not well understood, certain cytokines obtained from inflammation sites, particularly from extrahepatic adipose tissue, have been reported to induce this process [15]. Hepatic de novo lipogenesis is also known to be a unique feature in steatosis [15]. Insulin resistance has also been reported to lead to metabolomic dysregulation in NAFLD that activates and aggravates hepatic steatosis [13]. In total, 20–30% of NAFLD patients with simple steatosis progress to NASH [13].

In this study, we analyse various types of omics datasets, such as transcriptomics and lipidomics, in an effort to gain a better understanding of the NAFLD’s underlying pathophysiologic processes. Initially, we analysed transcriptomics data to identify potential gene biomarkers involved in the development of NAFLD and then proceeded to analysing lipidomics data so as to identify potential lipid biomarkers, as well as the pathways which are perturbed by these biomarkers.

## 2. Materials and Methods

**Figure 1:**
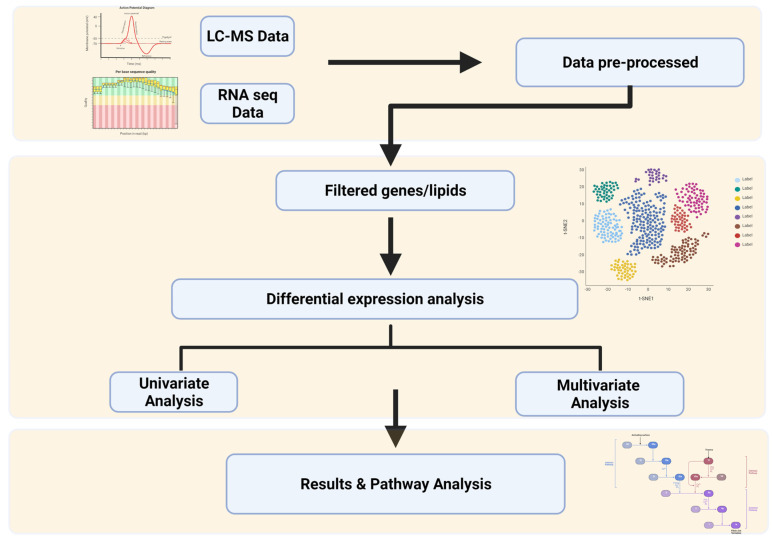
(Created with BioRender.com) NAFLD biomarkers identification study design consisting of pre-processing step followed by differential expression analysis for feature selection and then univariate and multivariate analysis. The final step includes the results interpretation and pathway analysis.

The schematic diagram presents the pipeline developed for the biomarker [16,17] identification using NAFLD-related transcriptomics and lipidomics datasets (Figure 1)**.**

### 2.1. Transcriptomics

#### 2.1.1. Data Acquisition

The datasets, GSE151158, GSE58979, GSE63067, GSE89632 and GSE33814, employed in this study were downloaded from the Gene Expression Omnibus (GEO) repository on 21 January 2021. In total, these datasets consisted of 146 samples, 81 of which were steatosis-related and 65 were control. The data were split into training, testing and validation sets and were subjected to pre-processing, normalization, data integration, batch-effect correction, PCA analysis, differential gene expression analysis, identification of common significant genes, as well as supervised analysis using random forest and biological interpretation.

Each GEO dataset was downloaded and loaded into R (version 4.0.3) by using the getGEO function in the *GEOquery* package (version 2.58.0) [18]. All datasets, apart from GSE151158, were already normalised. GSE151158 was normalised using the *edgeR* package (version 3.32.1) [19] *cpm* function with a True log parameter.

#### 2.1.2. Derivation of New Transcriptomics Cohort from Multiple GEO Datasets

Due to the number of control and steatosis samples, in each GEO dataset, being low (Table 1), GSE151158, GSE58979, GSE63067 and GSE89632 were integrated to derive a transcriptomics cohort that can be used for the downstream analysis, while GSE33814 was kept for validation. Following the datasets’ integration, in the derived cohorts, batch effects were identified using PCA (principle component analysis) plots. The PCs were generated using the function *prcomp* in the *stats* package and the PCA plots were visualized using *ggbiplot* (version 0.55) [20]. The batch correction for the derived cohort was performed based on non-parametric adjustment using *ComBat* [21], where batch effects due to different sequencing platforms were corrected. Following batch correction, PCA was performed to cater their visualisation.

#### 2.1.3. Differential Gene Expression (DGE) Analysis

The differential gene expression (DGE) analysis was performed using the *lmFit* and *eBayes* functions, available within the *limma* package (version 3.46.0) [27]. The application of the Benjamini–Hochberg (BH) correction method yielded a gene table consisting of the log fold change (logFC) and the adjusted *p* value. Significant genes with an adjusted *p* value less than 0.05 were then extracted. This gene list was further filtered to only include genes which were common between the derived cohort and validation set.

#### 2.1.4. Random Forest-Based Predictions

The derived cohort was split into a testing (control, 16; steatosis, 27) and a training set (control, 36; steatosis, 64). The validation set consisted of 13 control and 19 steatosis samples.

The random forest was set for repeated 10-fold cross validation with 5 repeats. The parameters of the random forest were tuned by the *expand.grid* method for the factors *mtry*, (12), *ntree* (55) and *maxnode* (6) using the training data and *train* function from the *caret* package (version 6.0.86) [28]. The model was then tested and validated using the test and validation set.

The accuracy of the model in classifying the steatosis samples for the testing and validation sets was evaluated based on the receiver operating characteristics (ROC) analysis. The area under the curve (AUC), sensitivity and specificity for both datasets were calculated by using the *pROC* package (version 1.17.0.1) [29].

#### 2.1.5. Downstream Data Analysis

The Wilcoxon test was conducted on the training and validation datasets to investigate the genes which were upregulated and downregulated between the control and the steatosis. A further pathway and GO enrichment analysis was performed using *enrichR* (version 3.0) [30]. The correlation plot for the genes in the training dataset was plotted using the package *corrplot* (version 0.84) [31].

### 2.2. Lipidomics Data Analysis

#### 2.2.1. Data Acquisition

Two cohorts (the Fenland cohort and the Italian cohort) were collected from Sanders et al., for identifying the biomarkers for NAFLD [32]. Both the cohorts consisted of clinical data and lipidomics data.

#### 2.2.2. Data Pre-Processing

The dataset was loaded into R (version 4.0.3) and was separated into clinical data and lipid expression data. Each of the expression data row was annotated according to the lipid names. The dataset was then scaled and the values of the missing features were imputed according to the feature mean.

#### 2.2.3. Differential Lipid Expression Analysis

A differential lipid expression analysis was performed on the Italian cohort using the *lmFit* and *eBayes* functions present in the *limma* package (version 3.46.0) [27]. The sample number difference, for each sample type, resulted in a class imbalance for the Italian cohort where there were 120 samples in steatosis0 and 21 samples in steatosis1. To address this, the steatosis0 samples were separated into 6 different batches, each containing 20 steatosis0 samples, ensuring that the samples in each of the 6 batches were unique and not repeated in other batches. The topTable function in *limma* obtained Benjamini–Hochberg (BH)-corrected *p* values and the logFC change of significant lipids between the steatosis0 and steatosis1 samples. A Volcano plot was plotted using the *ggplot2* (version 3.3.3) [33] for all the 6 different batches to visualize the differentially expressed lipids. The common significant lipids among these 6 different batches were obtained and were further subjected to a differential expression analysis using the 120 steatosis0 and 21 steatosis1 samples to obtain their logFC and *p* values.

#### 2.2.4. Random Forest and ROC Curve Analysis

The first random forest model was formed using the Italian cohort as the training set and the Fenland data as the test set. A stratified k fold cross validation approach was implemented, where the fold value was set to 5. The parameters of the random forest were tuned by using *expand.grid* for the factors *mtry* (12), *ntree* (150) and *maxnode* (6) using the training data and *train* function from *caret* package (version 6.0.86) [28]. The model was then tested and validated using the test and validation set.

Then, a second random forest model was generated using the Fenland data as the training set and the Italian cohort as the test set. A stratified k fold cross validation approach was implemented, where the fold value was set to 5. The parameters of the random forest were tuned by *expand.grid* for the factors *mtry* (2), *ntree* (55) and *maxnode* (6) using the training data and *train* function from *caret* package (version 6.0.86) [28]. The model was then tested and validated using the test and validation set.

The AUC, sensitivity and specificity for both the models was calculated by using the *pROC* package (version 1.17.0.1) [29].

### 2.3. Statistical Analysis

A Wilcoxon test was conducted using the *rstatix* package (version 0.7.0) [34]. Since the samples were unpaired, the wilcoxon test paired option was set to false and the confidence level was set to 0.95. The *p* values from this test, for each of the significant features, were extracted and stored in a new data frame. A boxplot was then generated to understand the significant difference between the steatosis0 and steatosis1 samples.

#### 2.3.1. Heatmap-Based Visualization of Significant Lipid Features

Two heatmap were created using the *ComplexHeatmap* (version 2.6.2) [35] package using 7 significant lipids. Heatmap 1 consisted of the 120 steatosis0 samples, while heatmap 2 was formed using the 21 steatosis1 samples. These two heatmaps were further combined to construct the complex heatmap that represented both the steatosis0 and steatosis1 samples variation across the lipids. The row title of the heatmap was set to the lipid names and the columns of the heatmap represents the steatosis0 and steatosis1 sample IDs.

#### 2.3.2. LIPEA-Based Lipid Pathway Enrichment Analysis

The LIPEA lipid pathway enrichment analysis [36] employs the lipid compounds IDs contained in the KEGG Database (Kyto Encyclopedia of Genes and Genomes) and identifies significantly disrupted pathways by applying a Fisher’s exact test followed by an over representation analysis (ORA) for each pathway; an output table, consisting of the enriched pathways, the lipids involved in them and their *p* values, is then generated.

#### 2.3.3. Lipid Network Analysis

A network analysis was performed to obtain potential interactions between the various lipid classes. The R *qgraph* package (version 1.6.9) [37] was used to generate the network, using the 7 significant lipids, with nodes, representing lipids, connected to weighted edges resembling the interaction between them. A Benjamini–Hochberg correction was implemented and the significant threshold was set to 0.05.

## 3. Results

### 3.1. Feature Selection for Random Forest Model

#### 3.1.1. Gene Signature Identification

**Figure 2:**
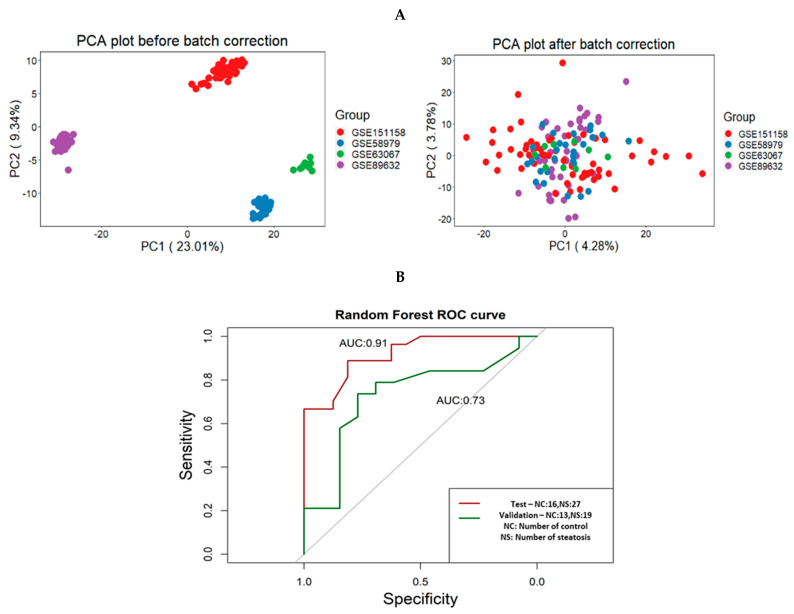
(**A**) A PCA plot that shows how the datasets behaved before and after the batch correction was implemented using *ComBat*. (**B**) The AUC values of the test and validation sets of the random forest model.

By merging GSE151158, GSE58979, GSE63067 and GSE89632, new NAFLD transcriptomics data were derived. Figure 2A depicts the PCA (principle component analysis) for the newly derived cohort before and after batch correction. To identify significant genes, a differential gene expression analysis was conducted over the derived transcriptomics datasets. In the derived transcriptomics cohort training data (GSE151158, GSE58979, GSE63067 and GSE89632), 173 genes were identified as significant (126 were upregulated and 47 were downregulated). Within the validation set (GSE33814), there were 1971 significant genes (772 were upregulated and 1199 were downregulated). Between the training and the validation sets, there were 18 common significant genes (C9, HPRT1, TLR1, B2M, BAX, GAPDH, BTK, PTPN6, SERPING1, ITGAE, IL1RAP, MSR1, TNFRSF14, IL15, CX3CR1, TOLLIP, IFIH1 and C4BPA) forming the group that was used within the training and validation sets.

#### 3.1.2. Lipid Signature Identification

There were two lipid datasets, the Italian cohort and the Fenland cohort. Within the Italian cohort, 120 steatosis0 and 21 steatosis1 samples were present, indicating a class imbalance. The steatosis0 samples were separated into six batches and a differential expression analysis was conducted six different times using *limma*. A Benjamini–Hochberg (BH) correction was implemented on the differential lipids and the lipids with adjusted *p* value lesser than 0.05 were extracted. The number of significant lipids identified for the different batches were as follows: batch 1, 191; batch 2, 189; batch 3, 171; batch 4, 129; batch 5, 42; batch 6, 57. There were 11 significant lipids which were identified in all six batches (Cholesterol,CE(16:0),DG(34:1),DG(36:2),TG(52:2),TG(52:3),TG(53:2),TG(53:3), TG(53:6),TG(53:7),TG(54:2)) and, of those 11 significant lipids, 2 lipids (Cholesterol, CE(16:0)) were upregulated and the remaining 9 lipids were downregulated (DG(34:1), DG(36:2),TG(52:2),TG(52:3),TG(53:2),TG(53:3),TG(53:6),TG(53:7) and TG(54:2)) in the steatosis0 vs. steatosis1 samples.

### 3.2. Random Forest Model Performance

#### 3.2.1. Transcriptomic Features Analysis

The area under the receiver operating characteristic curve (AUC) was calculated for the random forest model to be 0.91. The prediction accuracy decreased within the validation data, with an AUC value of 0.73 (Figure 2B). We hypothesise that this prediction accuracy reduction might be a result of the reduced sample number.

#### 3.2.2. Lipidomic Features Analysis

There were seven (Cholesterol, CE(16:0), DG(36:2), TG(52:2), TG(52:3), TG(53:2) and TG(54:2)) lipids common to the and Italian and Fenland cohorts. These lipids were part of the first random forest model. The Italian cohort was split into training and testing sets and the Fenland cohort was kept as the validation set. There were 36 steatosis0 and 6 steatosis1 samples in the test set and 633 steatosis0 and 222 steatosis1 in the validation set.

**Figure 3:**
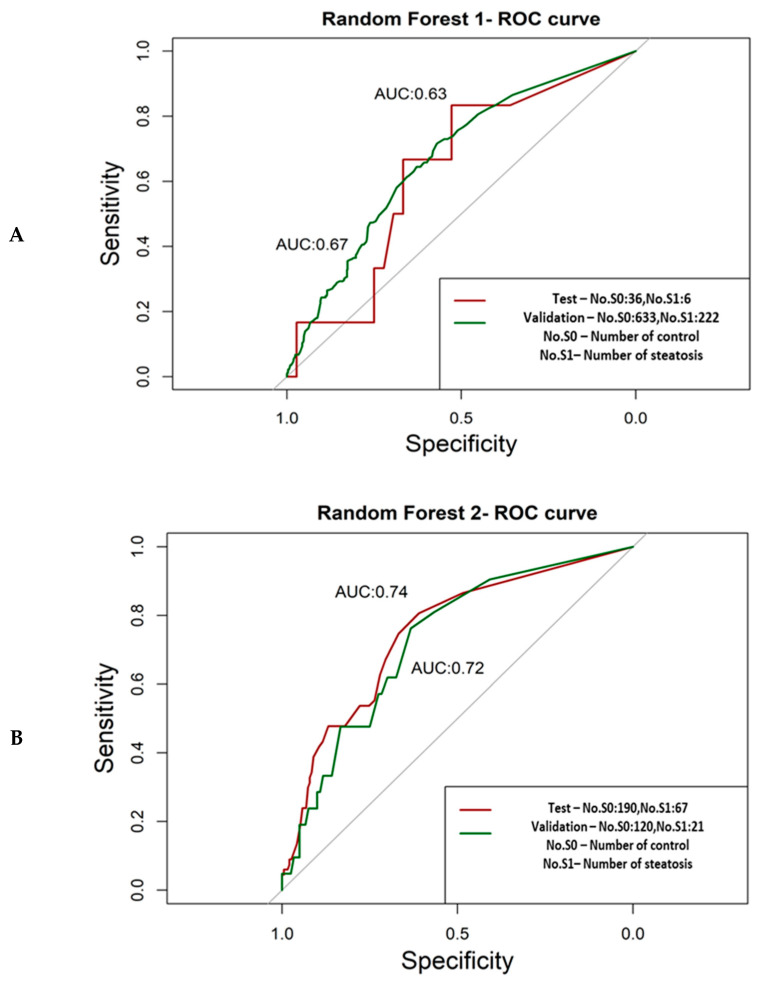
(**A**) Random forest 1 ROC curve that was formed using the Fenland cohort as the validation set. (**B**) Random forest 2 ROC curve that was formed using the Italian cohort as the validation set.

The resulting AUC value of random forest 1 for the test dataset is 0.63. An accuracy increase was reported for the validation dataset, with an AUC value of 0.67 (Figure 3A).

The process was repeated by alternating the Italian cohort as the validation set and the Fenland cohort as the training and testing set. There were 190 steatosis0 and 67 steatosis1 samples in the test set and 120 steatosis0 and 21 steatosis1 in the validation set.

Once the parameters were tuned by using the training set, the second random forest model was validated using the test and validation set. The AUC values for the test and validation datasets are 0.74 and 0.72, respectively (Figure 3B).

### 3.3. Downstream Analysis

#### 3.3.1. Transcriptomic Feature Study

**Figure 4:**
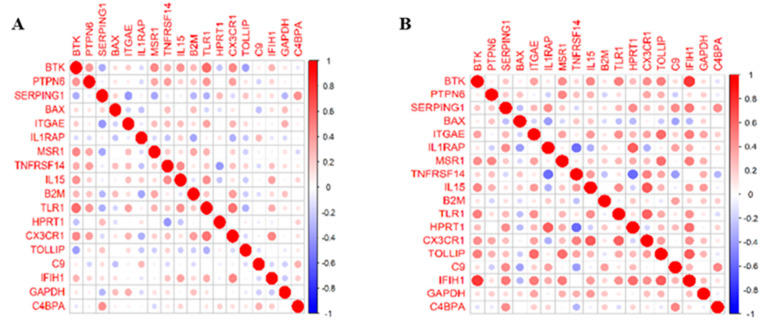
(**A**) A correlation gene matrixof control samples within the training dataset. (**B**) A correlation matrix of steatosis samples within the training dataset.

A pairwise correlation among 18 genes, within the training dataset, was identified within the control and the steatosis samples (Figure 4). The colour determines the sign of the coefficient, where the red colour represents a positive effect and the blue colour indicates a negative one (Figure 4A,B). The intensity of the colour increases proportionally to the magnitude of the correlation coefficient among the genes. When compared to the gene correlation matrix in the steatosis and control samples within the training data, the IFIH1 gene is positively correlated to BTK in the steatosis samples.

**Figure 5:**
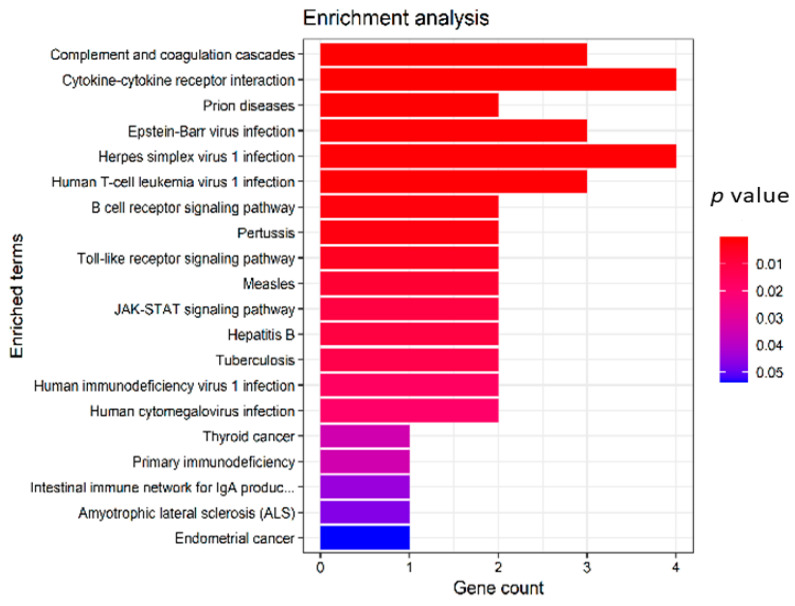
Enrichment analysis of gene signatures. Legend represents the *p* values associated with the pathways.

**Figure 6:**
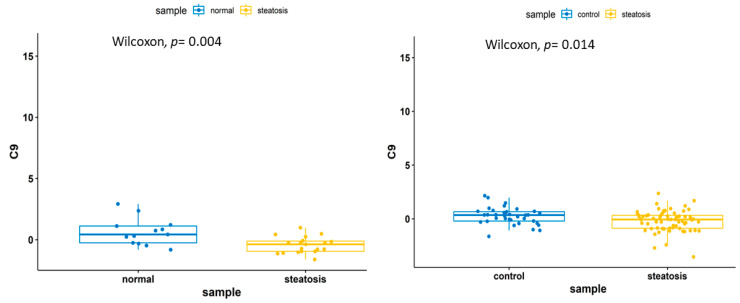
A boxplot denoting the downregulation of the C9 gene in the steatosis samples.

**Figure 7:**
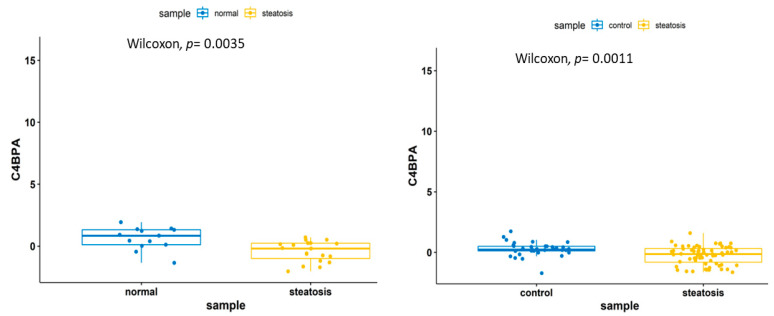
A boxplot showing the downregulation of the C4BPA gene in the steatosis samples.

A pathway enrichment analysis of the transcriptomics data identified the following pathways: complement and coagulation cascades (C9, SERPING1 and C4BPA), cytokine-cytokine receptor interaction (CX3CR1, IL15, TNFRSF14 and IL1RAP), herpes simplex virus 1 infection (IFIH1, BAX, TNFRSF14 and B2M), B cell receptor signaling pathway (BTK and PTPN6), Toll-like receptor signaling pathway (TLR1 and TOLLIP), JAK–STAT pathway (IL15 and PTPN6) and Human immunodeficiency virus 1 infection and primary immunodeficiency pathways (BAX and B2M) (Figure 5). Among the 18 genes within the training and the validation sets, 5 genes (HPRT1, C9, C4BPA, IL1RAP and TNFRSF14) were upregulated in both training and validation sets, whereas the other 13 genes were inconsistent between the training and the validation samples. Five genes, namely, IL1RAP, TOLLIP, HPRT1, C9 and C4BPA, have been previously identified to be in relation with NAFLD or other inflammatory responses [38,39]. A Wilcoxon test on C9 and C4BPA genes revealed gene downregulation within the steatosis samples (Figure 6 and Figure 7).

#### 3.3.2. Lipidomics Feature Study

**Figure 8:**
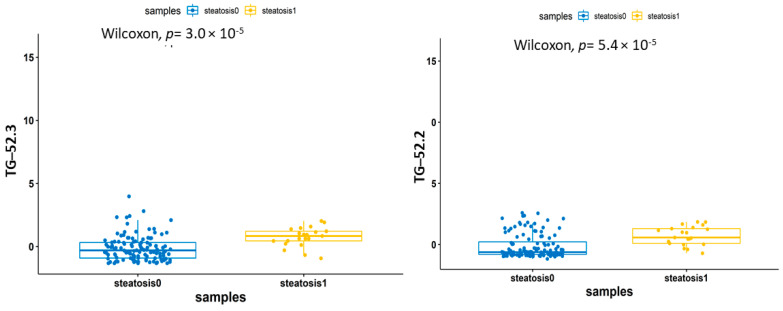
A boxplot denoting the upregulation of different lipid classes in steatosis1 when compared to steatosis0. The *p* value obtained from the Wilxocon test is significant.

The seven significant lipids, employed in the random forest model, were subjected to a Wilcoxon nonparametric statistical test and their corresponding *p* values were plotted. Among the seven lipids, TG (52.3) had the highest −log10 (*p* value). Further boxplots were generated based on these lipids, revealing that triglycerides were upregulated in the steatosis1 samples and downregulated in the steatosis0 samples (Figure 8).

**Figure 9:**
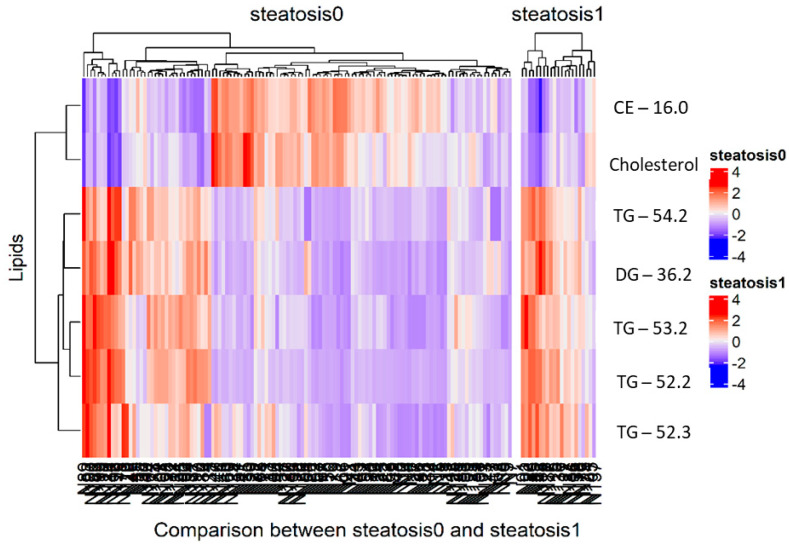
A complex heatmap consisting of steatosis0 and steatosis1 samples. Colours depict whether the lipids are positively or negatively correlated to the sample.

**Figure 10:**
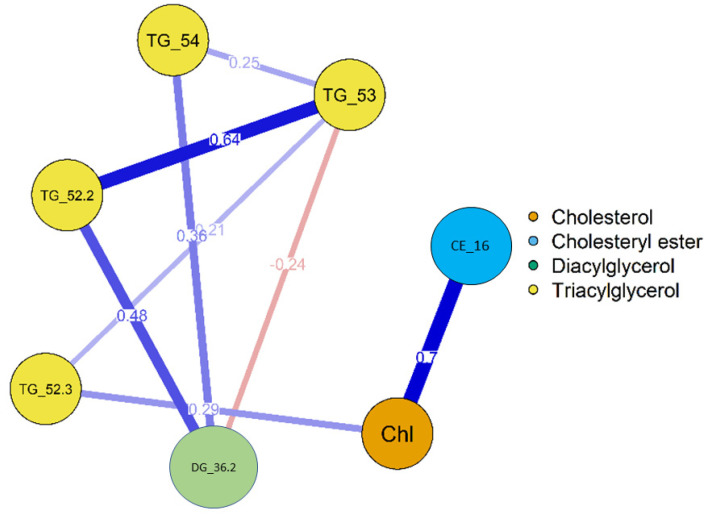
A lipid network showing the interactions among various groups. The blue colour indicates a positive correlation, while the red colour indicates a negative correlation.

A heatmap was constructed for the seven lipids across the steatosis0 and steatosis1 samples. The heatmap colours represent the coefficient signs and, more specifically, the red colour represents a positive effect and the blue colour indicates a negative effect. The majority of the steatosis0 samples exhibit a negative correlation effect on the triglycerides, whereas the steatosis1 samples had a positive correlation, supporting the notion that triglyceride upregulation could indicate NAFLD development (Figure 9). The lipid network shows that most of the triglycerides were positively correlated with one another (Figure 10).

A pathway enrichment analysis of the lipids revealed long-term depression, lipolysis in adipocytes, glycerolipid metabolism and insulin resistance as the most significant pathways in which those lipids are involved in (Table 2) (38).

## 4. Discussion

The NAFLD pathogenesis is complex and unhealthy lifestyle trends have substantially increased its health burden over the past few years [11,13]. Omics integrative analytics have been proposed as a promising approach to gain a better understanding of NAFLD’s biological underpinnings [40]. In this study, we analysed publicly available transcriptomic datasets to identify potential novel gene biomarkers, as well as the pathways which are perturbed.

The biomarkers identified by our transcriptomics analysis are primarily involved in immune-related pathways. Previous research studies have shown that NAFLD is related to an excessive activation of the immune system [41]. C4BPA, one of the genes identified by our transcriptomics analysis of the NAFLD samples, is primarily involved in immune-related pathways and has been identified as a target by several disease studies [42,43,44]. Research work has been conducted to study the defense function of C4BP against Influenza A Virus (IAV), an upper respiratory tract infection caused by the Influenza virus under the Orthomyxoviridae family which is known to cause the pandemic [45]. The complement system is safeguarded by various regulatory proteins, C4BP being one such humoral regulator example, to avoid unnecessary inflammation events [46]. Furthermore, C4BP-IgM has also been suggested as a target for the treatment of gonorrhea [47]. C9 and C4BPA have been further identified as key genes involved in the NAFLD development [38]. Moreover, IL1RAP and TOLLIP, involved in cytokine–cytokine interaction and Toll-like receptor signaling pathways, have been reported to play a key role in liver inflammatory diseases [39,48,49].

The complement system pathway is poorly characterised in NAFLD and NASH [50]. It is indicated that the complement system can be activated by three different pathways: the classical, the alternative and the lectin pathways [50]. The gene biomarker identified in our study is present in the complement and coagulation cascade pathway. C4BPA, one of the genes that was identified in this pathway, is also known as C4BP. It is indicated that C4BP is the main soluble inhibitor of the classical and the lectin pathways [51,52,53]. If the classical and lectin pathways are activated, they lead to the formation of c3 and c5 convertase and c3 and c5 conversions are central reaction in the complement activation.

The activation of the classical and lectin pathways leads to the apoptosis of hepatocytes which, in turn, renders the lipid metabolism; the improvement in controlling the apoptosis process might help in controlling NASH [54].

It is indicated that there is an increased level of c3 in obese individuals and the action of c3 convertase produces c3a and c3b. c3a has a short half life but is later converted into desArg c3a which has a longer half life [55]. desArg c3a, also known as ASP (acylation-stimulating protein), is involved in the increase in triglycerides in the plasma by causing ASP resistance. Metabolic resistance has also been indicated to be shared between insulin and ASP, where the increase in insulin levels might be caused by obesity [56].

Our differential lipid expression analysis identified 11 significant lipids. The most common class of those lipids was triglycerides. We observed a triglyceride upregulation between the steatosis0 and steatosis1, which is in agreement with recent reports of hypertriglyceridemia being common in NAFLD patients [57,58,59]. Due to the reason of over-nutrition or insulin resistance, triglyceride concentration within the liver becomes rendered and that might create an increase in the concentration of hepatic triglycerides, which leads to steatosis [58]. It is essential that the triglycerides are exported from the liver in the form of VLDL; if this process is affected, it results in steatosis [60]. Furthermore, the network construction has shown the positive correlation of triglycerides to the other lipid groups, cholesterol and diacylglycerol.

The pathways associated with the identified significant lipids are involved in adipocyte lipolysis. It has been previously reported that elevated body mass causes fat cell lipolysis [61]. This might further cause adipose tissue inflammation, which contributes to insulin resistance. Another function of insulin is to limit lipolysis by inhibiting HSL (hormone-sensitive lipase) [62].

In conclusion, our findings suggest that the downregulation of C4BP results in an activation of the lectin pathway in the complement system triggering the conversion of c3 to c3a and c3b by the action of c3 convertase, thereby increasing the triglyceride levels, as shown in our study. This indicates that C4BP could be a potential biomarker linked to the complement system pathway, that would aid in the treatment of NAFLD.

This study has several limitations. Firstly, a small number of transcriptomics samples were used to train and validate the model. Then, the dataset merging using *ComBat* might have led to the loss of information. Further studies with large sample sizes should be further conducted to validate our findings.

## 5. Conclusions

We identified C4BPA, which activates the complement and coagulation pathway that renders lipid metabolism, as a potential NAFLD biomarker.

## Figures and Tables

**Table 1:** Details of the transcriptomics data used in the study.

S.No	GEO	Number of Samples	Number of Features	Platform	Reference
1	GSE89632	Control (*n* = 24) vs. Steatosis (*n* = 20)	29,377	Illumina HumanHT-12 WG-DASL V4.0 R2 expression beadchip	[22]
2	GSE151158	Control (*n* = 21) vs. Steatosis (*n* =23)	618	NanoString Human Immunology v2 Code Set (NS_Immunology_v2_C2328+PLS_Golden_1_C5164)	[23]
3	GSE58979	Control (*n* = 0) vs. Steatosis (*n* = 17)	49,395	Affymetrix Human Gene Expression Array	[24]
4	GSE63067	Control (*n* = 7) vs. Steatosis (*n* = 2)	54,675	[HG-U133_Plus_2] Affymetrix Human Genome U133 Plus 2.0 Array	[25]
5	GSE33814	Control (*n* = 13) vs. Steatosis (*n* = 19)	48,803	Illumina HumanWG-6 v3.0 expression beadchip	[26]

**Table 2:** Pathway enrichment analysis of the significant lipids and their *p* values.

Pathway Name	Pathway Lipids	Converted Lipids (Number)	Converted Lipids (Percentage)	Converted Lipids (List)	*p*-Value
Long-term depression	3	2	50.00	C00165, C00641	0.0147783
Regulation of lipolysis in adipocytes	6	2	50.00	C00165, C00422	0.0147783
Glycerolipid metabolism	15	2	50.00	C00422, C00641	0.0421456
Insulin resistance	4	2	50.00	C00165, C00422	0.0421456
Fat digestion and absorption	8	2	50.00	C00165, C00422	0.0800387
Rap1 signaling pathway	1	1	25.00	C00165	0.137931
Chemokines signaling pathway	2	1	25.00	C00165	0.137931
Ras signaling pathway	2	1	25.00	C00165	0.137931
MAPK signaling pathway	1	1	25.00	C00165	0.137931
NF-kappa B signaling pathway	1	1	25.00	C00165	0.137931

Abbreviations: NAFLD, non-alcoholic fatty liver disease; ROC, receiver operating characteristics; PCA, principal component analysis; LIPEA, lipid pathway enrichment analysis; NASH, non-alcoholic steatohepatitis; GEO, Gene Expression Omnibus.

## Data Availability

The rmarkdown scripts and the html reports are available at: https://github.com/Roshanshafiha/NAFLD-Multi-Omics-Data-Analysis, accessed on 15 March 2021.
